# Centralized allocation of decision rights and enterprise transformation and upgrading: Based on human capital level and capital allocation efficiency

**DOI:** 10.1371/journal.pone.0319063

**Published:** 2025-03-17

**Authors:** Qingliu Tang, Hongfeng Sun

**Affiliations:** School of Economics and Management, Shihezi University, Shihezi, China; Adana Alparslan Turkes Science and Technology University: Adana Alparslan Turkes Bilim ve Teknoloji Universitesi, TÜRKIYE

## Abstract

Under the pressure of transformation and upgrading caused by complex environments at home and abroad, it is significant to explore how the centralized allocation of decision rights affects enterprise transformation and upgrading. Employing the data of Chinese A-share listed companies from 2010 to 2021, we demonstrate that the centralized allocation of decision rights significantly promotes enterprise transformation and upgrading. Further analysis indicates that enhanced human capital level and capital allocation efficiency are potential mechanisms through which centralized allocation of decision rights affects enterprise transformation and upgrading. Moreover, we document that the above positive correlation is more evident for groups with a high matching between cash flow rights and control rights, groups with strong supervision of major shareholders, and groups with a strong willingness of subsidiaries to cooperate. Collectively, these findings confirm the governance advantages of the centralized allocation of decision rights and provide important implications for enterprises’ transformation and upgrading in emerging market countries.

## 1. Introduction

In recent years, global economic development has faced severe challenges such as slowing growth, overcapacity, environmental pollution, and the COVID-19 shock, which makes enterprises face increasing pressure for transformation and upgrading [1,2]. For example, polluting enterprises need to achieve green transformation through environmental investment and green innovation to cope with ecological pollution pressure [3,4], and traditional manufacturing enterprises are in urgent need of intelligent and digital transformation to cope with the impact of the COVID-19 epidemic [5]. Notably, micro-level studies on the factors affecting enterprise transformation and upgrading primarily focus on government subsidies and credit intervention [6, 7], technological innovation [8, 9], environmental regulation [10], digital innovation [11], and entrepreneurial characteristics [12,13]. However, there is a lack of research to explore the role of enterprise groups’ centralized allocation of decision rights in enterprise transformation and upgrading.

Centralized allocation of decision rights of enterprise group refers to the organizational management mode of concentrating the decision rights of personnel, finance, R&D and other aspects on the parent company. There is mixed empirical evidence concerning the effect of the centralized allocation of decision rights. Some research demonstrates that centralized allocation of decision rights may cause serious agency problems [14], increase the cost of knowledge transfer [15], and trigger inefficient investment [16], thus causing stock price crash risk and damaging company value [17,18]. Other research identifies that centralized allocation of decision rights would alleviate agency problems [19], improve enterprise efficiency [20], reduce enterprise decision-making costs [21], restrain resource misallocation [22], and promote enterprise innovation [23]. However, the above literature primarily focuses on the institutional background of developed countries, and scarce research thus far has focused on the effect of the centralized allocation of decision rights on enterprise transformation and upgrading. In this context, we mainly investigate the influence of centralized allocation of decision rights on enterprise transformation and upgrading in emerging economies.

As the largest developing country, China provides an ideal scenario for examining the above issue. First and foremost, enterprise groups composed of several legally independent enterprises have become a typical pattern of enterprise organization in emerging market countries [23], especially in China, where more than 67.1% of public firms control at least one subsidiary. This means that a form of “one control many” group system is widespread in China, and most Chinese enterprise groups adopt the management mode of centralized allocation of decision rights [24]. That is, enterprise groups concentrate decision rights on the parent company [25]. In China’s economic system, authority and hierarchy are often obvious. Managers of enterprises in China usually have strong control rights and mainly implement a centralized management mode, while Western enterprises advocate a flat management mode more. In particular, the state-owned enterprises in which the Chinese government holds the controlling stake are the key components of the enterprise structure in China, accounting for a relatively high proportion. The commercial and public welfare characteristics of such enterprises are important attributes that distinguish Chinese enterprises from Western enterprises. The decision-making power of state-owned enterprise groups is centralized in the parent company, and unified management is implemented, which makes it easier to achieve the national economic and social goals. For example, state-owned enterprises need to promote the transformation and upgrading of enterprises and then drive the upgrading of the entire industrial structure to ensure that their development direction is consistent with national policies and plans. Second, China’s economic development begins to transition to a green, sustainable, high-quality model [3,4], which has made it difficult for the traditional crude development mode to fulfil the demands of economic development. Consequently, enterprises’ production and operation situation has become extremely complicated and rigorous, and enterprises urgently need to deal with the increasingly serious challenges through transformation and upgrading.

Adopting a sample of China’s A-share public companies from 2010 to 2021, we demonstrate that the centralized allocation of decision rights significantly promotes enterprise transformation and upgrading. Our results remain unchanged after a battery of robustness checks. Potential mechanism analysis shows that enhanced human capital level and capital allocation efficiency are two mechanisms through which centralized allocation of decision rights affects enterprise transformation and upgrading. In addition, we find that the above promotion relationship is more evident for groups with a high matching between cash flow rights and control rights, groups with strong supervision of major shareholders, and groups with a strong willingness of subsidiaries to cooperate.

The contributions of this study are mainly as follows: First, we contribute to the studies demonstrating the economic significance of the centralized allocation of decision rights. Extant research primarily examines the role of the allocation of decision rights in agency problems [19], enterprise efficiency [20], enterprise decision-making costs [21], resource misallocation [22], and enterprise innovation [23]. We supplement this literature by offering empirical evidence that the centralized allocation of decision rights promotes enterprise transformation and upgrading, which reveals the governance advantages of the centralized allocation of decision rights. Second, we enrich the studies on the determinants of enterprise transformation and upgrading from the perspective of decision rights allocation. While prior micro-level studies on the determinants of enterprise transformation and upgrading primarily focus on government subsidies and credit intervention [6,7], technological innovation [8,9], environmental regulation [10], digital innovation [11] and entrepreneurial characteristics [12,13], we document that the centralized allocation of decision rights matters in enterprise transformation and upgrading. Third, different from previous studies, we systematically analyze how the centralized allocation of decision rights can promote the transformation and upgrading of enterprises by improving the level of human capital and the efficiency of capital allocation. In particular, although this study is similar to Lou and Zhu’s research [23] on centralized allocation of decision rights and enterprise innovation, there are also essential differences. First of all, although there is a certain correlation between the research variable enterprise innovation and enterprise transformation and upgrading, there are crucial differences in definition and scope. Secondly, Lou and Zhu’s research did not explore the theoretical mechanism in depth, but this paper made a detailed theoretical analysis and tested the mediating effect. Finally, although there are similarities in the selection of control variables between this paper and Lou and Zhu’s research, the research model of Lou and Zhu is a U-shaped relationship model, which is different from the linear relationship model in this paper.

## 2. Theoretical analysis and hypothesis development

According to the resource-configuration theory, heterogeneous resource elements produce different effects when they are configured, and enterprise transformation and upgrading should be the result of the optimal allocation of resource elements [26]. Numerous studies have shown that human capital level and capital allocation efficiency are the most essential resource elements affecting enterprise transformation and upgrading [27]. Therefore, from the perspective of element allocation, we analyze the effect of the centralized allocation of decision rights on enterprise transformation and upgrading in terms of human capital level and capital allocation efficiency.

### 2.1. Based on the perspective of human capital level

First, the centralized allocation of decision rights can enhance the human capital level by alleviating financing constraints, thus promoting enterprise transformation and upgrading. Broadly speaking, enterprise transformation and upgrading have a rigid demand for funds, while in the early stage of transformation, enterprises usually face greater risks and weak credit guarantees, resulting in the low willingness of external financing institutions to lend, thus aggravating enterprises’ financing constraints. Scholars represented by Williamson [28] argue that companies can alleviate financing constraints by structuring internal capital markets. Arguably, enterprise groups with a centralized allocation of decision rights can leverage the internal capital market to achieve complementarity of resources among group members, thus creating a resource integration effect [29]. Moreover, the centralized allocation of decision rights allows the group to adopt a centralized debt model to reduce loan risks to enhance external financing capabilities [30] and play a joint guarantee effect, thereby alleviating financing constraints. This will be expected to guarantee a stable salary and promotion plan for employees, allow the group to provide preferential policies or special treatment for outstanding talents, and supplement working capital to increase the share of labour income, thereby attracting talent aggregation. In addition, the alleviation of financing constraints can also unify the group’s employment policy and establish a perfect staff training system [20], further enhancing human capital levels to facilitate enterprise transformation and upgrading.

Second, the centralized allocation of decision rights can enhance the human capital level by promoting knowledge and information sharing, thus promoting enterprise transformation and upgrading. First, the centralized allocation of decision rights can use consistent strategic management to establish mandatory norms of sharing behavior, thereby reducing the cost of knowledge information transmission within the group and generating knowledge information sharing effects [31]. This can be reasonably expected to enrich and expand enterprises’ knowledge boundary, create opportunities and platforms for mutual learning, and promote better exchange and learning among talents [32], thus improving enterprises’ human capital quality and facilitating enterprise transformation and upgrading. Second, most knowledge is implicit and difficult to transmit, which is commonly called experience or intuition. The centralized allocation of decision rights gives the delegated personnel group authority so that they have higher power and motivation to utilize their own rich experience, knowledge, and ability to drive the talents’ growth within the group, thereby making implicit knowledge visible and generating spillover effects [33], and ultimately improving the quality of human capital in the enterprise groups, thereafter promoting enterprise transformation and upgrading.

### 2.2. Based on the perspective of capital allocation efficiency

First, the internal capital market constructed by centralized allocation of decision rights can not only alleviate the financing constraints of enterprises, but also revitalize funds and improve resource utilisation efficiency, thus promoting enterprises’ transformation and upgrading. Due to the limited resources of the group, the parent company will choose the “winner” among the subsidiaries to optimize the resource allocation [34]. This not only curbs the inefficient investment of subsidiaries, but also avoids the rent-seeking behavior of subsidiaries, thereby enhancing the group’s investment efficiency to promote enterprise transformation and upgrading.

Second, the centralized allocation of decision rights can establish a unified management system, such as a fair personnel system, a standard workflow, and a normative financial system, which will help reduce enterprises’ information transmission cost [21], improve management efficiency [20], and to a certain extent promote the efficient coordination among companies within the group, thus optimizing the group’s investment efficiency to promote enterprise transformation and upgrading.

Third, the centralized allocation of decision rights can improve the group authority of the parent company to appoint the head and optimize the allocation of resources to promote the transformation and upgrading of enterprises. Generally, the parent company usually appoints directors, supervisors, and senior administrators to exercise coordination, supervision, and appraisal functions over the subsidiary. At the same time, the parent company generally retains the right to appraise and make decisions on the salaries of delegated individuals [35], so as to avoid collusion between the appointed personnel and the subsidiaries and harm the interests of the company. When the decision rights of the enterprise groups are decentralized, the head appointed by the parent company tends to lack authority and show weaker governance capacity. In contrast, when the decision rights of the enterprise groups are centralized, the appointed person can take advantage of the group authority and give full play to the governance role. This can not only weaken the discretion of the subsidiary’s management and curb their opportunistic behavior but also enable them to know the subsidiary’s business activities, reducing information asymmetry between the parent and the subsidiary to improve investment efficiency [19], thereby promoting enterprise transformation and upgrading.

Taken together, given the foregoing arguments, we posit the following hypothesis:

H. The centralized allocation of decision rights significantly promotes enterprise transformation and upgrading.

## 3. Research methodology

### 3.1. Data

We select Chinese A-share public companies from 2010 to 2021 as the initial sample. Empirical data derives from the CSMAR database, WIND database, EPS database, and Juchao Information Network. Specifically, R&D expense data are derived from the WIND database. GDP, foreign direct investment, and financial development level data are downloaded from the EPS database. The data on the geographical distance between parent and subsidiary companies are hand-collected from the Juchao Information Network. Other financial and corporate governance data are retrieved from the CSMAR database. Referring to previous studies, we use the following criteria to clean the sample. First, we remove the samples marked as abnormal by the stock exchange. Second, we drop the samples of financial insurance, negative net assets, and no subsidiaries. Third, missing data samples are excluded. To attenuate the influence of outliers, we winsorize all continuous variables at the 1% level. After these steps, we obtained 19141 firm-year sample observations.

### 3.2. Models and variables

We use the following equation to examine the hypothesis:


$$TFP_{i,t}=\alpha_{0}+\alpha_{1}Cen_{i,t}+\alpha_{2}Controls_{i,t}+\sum Year+\sum Ind+\varepsilon_{i,t}$$ (1)

where the dependent variable TFP_i,t_ represents the enterprise transformation and upgrading. TFP_i,t_ is a very broad and vague concept. There are different methods of measuring TFP_i,t_, but a unified standard has not yet been established. Wen et al. [6] emphasize that total factor productivity combines technical level, management ability, and other factors, which can comprehensively measure the effect of enterprise transformation and upgrading. Therefore, following Wen et al. [6], we employ total factor productivity to measure enterprise transformation and upgrading. In particular, we adopt the OP method to calculate TFP_i,t_ [36].

The independent variable Cen_i,t_ denotes the centralized allocation of decision rights. According to Lou and Zhu [23], the employee remuneration payments by the enterprise group capture the parent company’s control over the group’s personnel rights; it further captures the parent company’s control over the group’s various decision rights. Hence, following previous studies [23,25], we construct the following equation (2) and regress it by year and industry, with the estimated residual as a measure of Cen_i,t_.


$${\text{PSalary}}_{i,t}\text{= }\beta_{0}\text{+}\beta_{1}{\text{PAsset}}_{i,t}\text{+}\sum Year+\sum Ind\text{+ }\varepsilon_{i,t}$$ (2)

where Psalary_i,t_ is the proportion of employee remuneration payments by the parent company. It is measured as the “Cash paid to and for employees” item in the parent company’s cash flow statement, scaled by the corresponding item in the consolidated statement. Passet_i,t_ is the percentage of the parent company’s assets, measured by dividing total parent company assets by total consolidated statement assets. Before the regression, PSalary_i,t_ and PAsset_i,t_ are tailed by the interval [0,1].

The control variables are taken from prior studies [6,23] involving company size (Size), asset-liability ratio (Lev), company age (Age), return of assets (Roa), cash flow (Cash), R&D intensity (Rd), independent director ratio (Indep), institutional shareholding ratio (Organ), regional economic level (GDP), foreign direct investment (FDI), financial development level (FIN). Simultaneously, industry and year-fixed effects are controlled, which is the same as the model (1). Detailed variable definitions are presented in Table 1.

**Table 1:** Variable definitions.

Variable	Definition	Data Source
TFP	Total factor productivity calculated by OP method	CSMAR Database
Cen	See model 2	CSMAR Database
Size	The natural logarithm of total assets	CSMAR Database
Lev	Total debt divided by total assets	CSMAR Database
Age	The natural logarithm of listed years of enterprises	CSMAR Database
Roa	Net profit divided by total assets	CSMAR Database
Cash	Net cash flow from operating activities divided by total assets	CSMAR Database
Rd	R&D expenditure scaled by assets	WIND Database
Indep	The ratio of the number of independent directors to board directors	CSMAR Database
Organ	The proportion of shares held by external institutional investors	CSMAR Database
GDP	The natural logarithm of the Per capita GDP of each province	EPS Database
FDI	The ratio of foreign direct investment to local GDP	EPS Database
FIN	The ratio of deposits and loans of financial institutions to local GDP	EPS Database

Note: These variables are total factor productivity (TFP), centralized allocation of decision rights (Cen), company size (Size), asset-liability ratio (Lev), company age (Age), return of assets (Roa), cash flow (Cash), R&D intensity (Rd), independent director ratio (Indep), institutional shareholding ratio (Organ), regional economic level (GDP), foreign direct investment (FDI), and financial development level (FIN).

## 4. Empirical results and discussions

### 4.1. Descriptive statistics

Table 2 gives the descriptive statistical results of key variables. The mean, standard deviation and maximum and minimum values of TFP indicate great differences in the transformation and upgrading levels of sample enterprises. The mean and standard deviation of Cen indicate great differences in the organizational management modes of enterprise groups. Because the median of Cen is greater than 0, more than half of the enterprise groups choose the management mode of centralized allocation of decision rights. Meanwhile, the descriptive statistical results of control variables are consistent with previous literature [23,25].

**Table 2:** Descriptive statistics for key variables.

Variables	Obs	Mean	S.D.	Minimum	Median	Maximum
TFP	19141	7.360	0.885	5.503	7.250	9.874
Cen	19141	-0.005	0.224	-0.629	0.005	0.463
Size	19141	22.139	1.258	19.155	21.948	26.150
Lev	19141	0.411	0.201	0.049	0.402	0.895
Age	19141	2.145	0.719	0.475	2.210	3.341
Roa	19141	0.040	0.059	-0.251	0.038	0.213
Cash	19141	0.005	0.088	-0.249	0.003	0.488
Rd	19141	0.025	0.024	0.000	0.021	0.145
Indep	19141	0.375	0.053	0.333	0.333	0.571
Organ	19141	0.428	0.248	0.003	0.448	0.918
GDP	19141	11.103	0.465	9.995	11.102	12.009
FDI	19141	0.024	0.013	0.001	0.022	0.096
FIN	19141	0.009	0.007	0.001	0.006	0.033

Note: This table presents summary statistics. The sample includes 19141 firm-year observations from 2010 to 2021. These variables are total factor productivity (TFP), centralized allocation of decision rights (Cen), company size (Size), asset-liability ratio (Lev), company age (Age), return of assets (Roa), cash flow (Cash), R&D intensity (Rd), independent director ratio (Indep), institutional shareholding ratio (Organ), regional economic level (GDP), foreign direct investment (FDI), and financial development level (FIN). Detailed definitions of these variables are given in Table 1.

### 4.2. Baseline regression results

Table 3 presents the estimation results of the baseline model. The main variable of focus in the model (1) is the centralized allocation of decision rights (Cen). Column (1) presents the basic effects of the centralized allocation of decision rights on enterprise transformation and upgrading while only controlling for company-level control variables. The coefficient on Cen is 0.112 and significantly positive, suggesting that the centralized allocation of decision rights promotes enterprise transformation and upgrading. Based on column (1), the control variables at the regional level are added, and the results are shown in column (2). The results show that the coefficient on Cen is 0.122 and significantly positive. Based on column (2), column (3) further adds the year-fixed effects and industry-fixed effects. The coefficient on Cen is still significantly positive at the 1% level. Collectively, these findings evidence that the centralized allocation of decision rights can facilitate enterprise transformation and upgrading, providing empirical support for the hypothesis.

**Table 3:** The centralized allocation of decision rights affects enterprise transformation and upgrading.

Variables	(1)	(2)	(3)
	TFP	TFP	TFP
Cen	0.112^***^	0.122^***^	0.119^***^
	(0.00)	(0.00)	(0.00)
Size	0.476^***^	0.461^***^	0.466^***^
	(0.00)	(0.00)	(0.00)
Lev	0.775^***^	0.833^***^	0.739^***^
	(0.00)	(0.00)	(0.00)
Age	0.043^***^	0.051^***^	0.046^***^
	(0.00)	(0.00)	(0.00)
Roa	2.186^***^	2.268^***^	2.045^***^
	(0.00)	(0.00)	(0.00)
Cash	-0.049	-0.077^**^	-0.078^**^
	(0.20)	(0.04)	(0.03)
Rd	0.197	-0.402	0.591
	(0.58)	(0.28)	(0.13)
Indep	-0.092	-0.152	-0.097
	(0.54)	(0.30)	(0.47)
Organ	0.013	0.048	0.037
	(0.72)	(0.19)	(0.29)
GDP		0.169^***^	0.148^***^
		(0.00)	(0.00)
FDI		1.100^*^	1.501^**^
		(0.08)	(0.02)
FIN		1.129	0.767
		(0.38)	(0.54)
cons	-3.654^***^	-5.263^***^	-5.130^***^
	(0.00)	(0.00)	(0.00)
N	19141	19141	19141
Ind/Year	NO	NO	YES
AR2	0.632	0.641	0.674

Note: The table shows that the explained variable is total factor productivity (TFP), the explanatory variable is centralized allocation of decision rights (Cen), and the other control variables are company size (Size), asset-liability ratio (Lev), company age (Age), return of assets (Roa), cash flow (Cash), R&D intensity (Rd), independent director ratio (Indep), institutional shareholding ratio (Organ), regional economic level (GDP), foreign direct investment (FDI), and financial development level (FIN). The control variables in the regression of column (1) only contain the control variables at the company level. Then, on this basis, column (2) continues to add control variables at the regional level. Finally, the year and industry fixed effects are further added in column (3). Robust standard errors are presented in parentheses. ***, **, and * denote statistical significance at the 1%, 5%, and 10% levels, respectively.

### 4.3. Robustness checks

#### 4.3.1. Instrumental variable approach.

To further mitigate the endogeneity issue, we employ 2SLS with instrumental variables for the test. Here, we mainly choose the geographical distance between parent-subsidiary companies. This is primarily because the farther the geographical distance between parent-subsidiary companies, the more serious the information asymmetry within the group [37], and the higher the cost of information communication and transmission, which increases the cost of group centralization and weakens the group’s willingness to centralize [15]. Therefore, we could believe that the geographical distance between parent-subsidiary companies will affect the group’s centralized allocation of decision rights, but will not directly affect enterprise transformation and upgrading. Further, we perform the 2SLS regressions as follows:


$${\text{Cen}}_{i,t}\text{=}\Gamma_{0}\text{+}\Gamma_{1}PGD_{i,t}\text{+ }\Gamma_{2}Controls_{i,t}\text{+}\sum Year\text{+}\sum Ind\text{+ }\varepsilon_{i,t}$$ (3)

$$TFP_{i,t}\text{=}{Ζ}_{0}\text{+}{Ζ}_{1}Cen\_PGD_{i,t}\text{+ }{Ζ}_{2}Controls_{i,t}\text{+}\sum Year\text{+}\sum Ind\text{+ }\varepsilon_{i,t}$$ (4)

where the model (3) is the first-stage estimation, and model (4) is the second-stage regression. PGD_i,t_ represents the instrumental variable; Cen_PGD_i,t_ is the fitted value of the first-stage estimation; other variables are the same as in model (1).

Columns (1) and (2) in Appendix 1 show the results of the instrumental variable approach. In the first-stage estimations in column (1), the instrumental variable PGD is statistically negatively correlated with Cen, suggesting that the instrumental variable meets the relevance criterion. Moreover, the F-statistics of the first-stage regression is 362.384 and significantly positive, thereby rejecting the weak instrumental variables hypothesis. Column (2) shows the second-stage results of TFP as the dependent variables. The coefficient on Cen_PGD is 0.595 and significantly positive, confirming the robustness of the aforementioned basic results.

#### 4.3.2. Propensity score matching (PSM).

Although we argue that there is a positive association between the centralized allocation of decision rights and enterprise transformation and upgrading, the benchmark findings may suffer from self-selection bias. That is, enterprise groups with specific features may be more inclined to adopt centralized management and prefer to carry out transformation and upgrading. To ease this concern, we re-regress the equation (1) employing the propensity score matching method.

First, our indicator variable measuring the group’s decision rights, Cen_H, equals one if the value of Cen is greater than zero, showing that the group’s decision rights are centralized; otherwise, Cen_H equals zero, indicating that the group’s decision rights are decentralized. Second, we regress our indicator variable Cen_H, employing a logit model to estimate the propensity score for a group to have a centralized allocation of decision rights. Afterwards, the treatment group sample (Cen_H = 1) is matched to the control group sample (Cen_H = 0), which has the closest propensity score. We require the caliper to be 0.05 and perform the matching with replacement. Appendix 2 details the results of the equilibrium hypothesis tests. It suggests the matching is overall well balanced as the differences across covariates are generally insignificant in all characteristics. Column (3) in Appendix 1 reports the regression results of model (1) with the matched sample. The coefficient on Cen is 0.053 and significantly positive, which is in line with the baseline findings in Table 3.

#### 4.3.3. Heckman selection model.

Since the propensity score matching method can only alleviate the self-selection bias problem of observable variables, we further use the Heckman selection model to solve the sample selection problem of unmeasurable variables [38].

In the first stage, we employ the probit model to estimate the probability that a group adopts centralized management, measured as the indicator variable, as noted in section 4.3.2. Referring to previous literature [25], we control the following variables: company size (Size), asset-liability ratio (Lev), firm age (Age), return of assets (Roa), cash flow (Cash), R&D intensity (Rd), independent director ratio (Indep), institutional shareholding ratio (Organ), The ratio of net fixed assets to total operating income (Zbcc), The ratio of the total assets of the parent company’s statements to the total assets of the consolidated statements (Asset_ratio), The ratio of the shareholding ratio of the 2nd to 5th largest shareholder to that of the largest shareholder (Balance), and managerial ownership (Mshare). Following Quan et al. [2], we adopt the industry annual average with the centralized allocation of decision rights (MCen) as the instrumental variable. We argue that enterprises in the same industry are likely to have incentives to adopt a similar allocation of decision rights, such an industry-level mean variable is likely to be positively correlated with Cen, but less likely to affect enterprise transformation and upgrading.

The first-stage regression results (Appendix 3) indicate that the coefficient on MCen is significantly positive. The Inverse Mills Ratio (IMR) generated in the first stage was subsequently incorporated into the second-stage regression to control for sample selection bias. Column (4) in Appendix 1 shows the second-stage results. The coefficient on Cen is 0.196 and remains significantly positive. Consequently, the above results indicate that our findings are unlikely due to sample selection bias.

In addition, we make a supplementary test of the Heckman selection model. First, we use the industry annual average with centralized allocation of decision rights (MCen) as the instrumental variable in the first stage of the Heckman selection model, and the control variables are consistent with the benchmark model (1). The regression results are shown in columns (1) and (2) of Appendix 4. Second, following the instrumental variable in Section 4.3.1, we use the geographical distance of the parent-subsidiary company (PGD) as an instrumental variable in the first stage of the Heckman selection model, and the control variables are consistent with the benchmark model (1). The regression results are shown in columns (3) and (4) of Appendix 4. Third, we delete the instrumental variable in the first stage regression of the Heckman selection model and then estimate the model. The regression results are shown in columns (5) and (6) of Appendix 4. These results are consistent with the expectations of the article.

#### 4.3.4. Using Cen with a lag of one year.

Considering that the impact of groups’ centralized allocation of decision rights on enterprise transformation and upgrading may have a certain time lag, thus we re-run our model (1) using Cen with a lag of one year (L.Cen). Column (5) in Appendix 1 shows the results. The coefficient on L.Cen is 0.151 and significantly positive, consistent with the above conclusions.

#### 4.3.5. Alternative measure for explained variables.

The level of innovation reflects a firm’s core competitiveness and is of great significance in promoting enterprise transformation and upgrading [9]. Hence, we use the level of innovation (Patent), measured as the natural logarithm of the number of patent applications, as an alternative variable for enterprise transformation and upgrading. Column (6) in Appendix 1 presents the results. The coefficient on Cen is 0.645 and significantly positive. Unsurprisingly, the result is in line with prior findings.

## 5. Further analysis

### 5.1. Mechanism test

By now, we have confirmed that the group’s centralized allocation of decision rights significantly promotes enterprise transformation and upgrading. Further, we explore the underlying mechanism behind the basic results in this section. According to the above analysis, human capital level and capital allocation efficiency are two important ways to influence enterprise transformation and upgrading [27], and the centralized allocation of decision rights can improve the corporate human capital level and capital allocation efficiency. To verify these mechanisms, we empirically test the above two channels.

First, referring to the research of Chemmanur et al. [39], we use the proportion of employees with bachelor’s degrees and above in the total number of employees to measure the human capital level (HCL). Second, referring to Richardson’s research [40]. We construct the following model (5) and estimate its residual, using the absolute value of the residual to measure enterprise inefficient investment (Absinv). Among them, the explained variable enterprise investment (Invest_i,t_) is the difference between the cash paid for the construction of fixed assets, intangible assets and other long-term assets and the net cash recovered from the disposal of fixed assets, intangible assets and other long-term assets, and divided by the total assets at the beginning of the period; the explanatory variables include company growth (Growth_i,t-1_), cash flow (Cash_i,t-1_), asset-liability ratio (Lev_i,t-1_), company age (Age_i,t-1_), annual stock return (Ret_i,t-1_), company size (Size_i,t-1_) and enterprise investment (Invest_i,t-1_), while controlling the year and industry fixed effects.


$$Invest_{i,t}\text{=}\mu_{0}\text{+}\mu_{1}Growth_{i,t-1}\text{+}\mu_{2}Cash_{i,t-1}\text{+}\mu_{3}Lev_{i,t-1}\text{+}\mu_{4}Age_{i,t-1}$$

$$\text{+}\mu_{5}Ret_{i,t-1}\text{+}\mu_{6}Size_{i,t-1}\text{+}\mu_{7}Invest_{i,t-1}\text{+}\sum Year\text{+}\sum Ind\text{+}\varepsilon_{i,t}$$ (5)

Table 4 presents the results. The explained variable is HCL in Panel A, and the control variables are taken from previous studies [41]. Specifically, we include company size (Size), asset-liability ratio (Lev), firm age (Age), cash flow (Cash), return of assets (Roa), independent director ratio (Indep), institutional shareholding ratio (Organ), and cash paid for long-term assets scaled by total assets (Capex). The coefficient on Cen is 2.357 and significantly positive, thereby providing evidence to support that the centralized allocation of decision rights facilitates enterprise transformation and upgrading by improving the human capital level.

**Table 4:** Potential mechanisms.

Panel A human capital level		Panel B capital allocation efficiency	
Variables	HCL	Variables	Absinv
Cen	2.357^***^	Cen	-0.006^***^
	(0.00)		(0.00)
Size	0.321^**^	Size	0.002^***^
	(0.04)		(0.00)
Lev	-6.953^***^	Lev	0.016^***^
	(0.00)		(0.00)
Age	0.590^**^	Age	0.001^***^
	(0.01)		(0.01)
Cash	-0.857	Cash	0.016^***^
	(0.60)		(0.00)
Roa	11.142^***^	Roa	0.029^***^
	(0.00)		(0.00)
Indep	13.583^***^	Indep	-0.002
	(0.00)		(0.80)
Organ	2.235^***^	Board	-0.008^***^
	(0.00)		(0.00)
Capex	-30.470^***^	Ci	0.002^***^
	(0.00)		(0.00)
cons	21.518^***^	cons	-0.009
	(0.00)		(0.19)
N	19141	N	19141
Ind/Year	YES	Ind/Year	YES
AR2	0.216	AR2	0.076

Note: Panel A and panel B of this table test the two mechanisms of human capital level and capital allocation efficiency, respectively. The explained variables are human capital level (HCL) and inefficient investment (Absinv), the explanatory variable is centralized allocation of decision rights (Cen), and the control variables are company size (Size), asset-liability ratio (Lev), firm age (Age), cash flow (Cash), return of assets (Roa), independent director ratio (Indep), board size (Board), institutional shareholding ratio (Organ), the ratio of total assets to total income (Ci) and cash paid for long-term assets scaled by total assets (Capex). Robust standard errors are presented in parentheses. ***, **, and *  denote statistical significance at the 1%, 5%, and 10% levels, respectively.

The explained variable is Absinv in Panel B, and the control variables are drawn from previous studies [42]. Specifically, we include company size (Size), asset-liability ratio (Lev), firm age (Age), cash flow (Cash), return of assets (Roa), independent director ratio (Indep), board size (Board), and the ratio of total assets to total income (Ci). The coefficient on Cen is -0.006 and significantly negative, thus providing empirical evidence that the centralized allocation of decision rights facilitates enterprise transformation and upgrading by improving capital allocation efficiency.

### 5.2. Cross-sectional analysis

#### 5.2.1. The matching degree of cash flow rights and control rights.

In emerging economies where investor protection is insufficient, the intra-conglomerate capital market, which was originally designed to improve the efficiency of capital allocation, has been partially alienated as a channel for transferring benefits to controlling shareholders. Broadly speaking, only when the matching degree between cash flow rights and control rights is high can the group restrain the parent company’s tunnelling motivation and minimize the resource dissipation caused by the centralized allocation of decision rights [43], thus giving full play to the governance advantages of the centralized allocation of decision rights. If so, it could be expected that the positive impact of the centralized allocation of decision rights on enterprise transformation and upgrading is more pronounced for groups with a high matching between cash flow rights and control rights. Following Chandler [44], we first standardize the group’s control rights (Cen) and cash flow rights (CashPower), and then use the matching formula PPD =  1- | Cen – CashPower | to obtain the matching degree of the group’s cash flow rights and control rights. Based on the model (1), the interaction term of Cen and PPD and the PPD variable are added, and the baseline model is re-estimated. Panel A in Table 5 presents the estimation results. The coefficient on the interaction term is 0.180 and significantly positive, confirming our expectation that the positive effect of the centralized allocation of decision rights on enterprise transformation and upgrading is more pronounced for groups with a high matching between cash flow rights and control rights.

**Table 5:** Cross-sectional tests.

Panel A the matching degree of cash flow rights and control rights		Panel B the supervision of major shareholders		Panel C the willingness of subsidiaries to cooperate	
Variables	TFP	Variables	TFP	Variables	TFP
Cen	0.134^***^	Cen	0.113^***^	Cen	0.230^***^
	(0.00)		(0.00)		(0.00)
Cen^*^PPD	0.180^***^	Cen^*^Top1	0.003^**^	Cen*SOP	0.253^***^
	(0.00)		(0.02)		(0.00)
PPD	0.043^***^	Top1	0.002^***^	SOP	0.167^***^
	(0.00)		(0.00)		(0.00)
Size	0.465^***^	Size	0.465^***^	Size	0.459^***^
	(0.00)		(0.00)		(0.00)
Lev	0.737^***^	Lev	0.741^***^	Lev	0.703^***^
	(0.00)		(0.00)		(0.00)
Age	0.044^***^	Age	0.053^***^	Age	0.034^***^
	(0.00)		(0.00)		(0.00)
Roa	2.054^***^	Roa	2.037^***^	Roa	2.008^***^
	(0.00)		(0.00)		(0.00)
Cash	-0.074^*^	Cash	-0.071^*^	Cash	-0.075^*^
	(0.08)		(0.10)		(0.08)
Rd	0.594^***^	Rd	0.676^***^	Rd	0.638^***^
	(0.00)		(0.00)		(0.00)
Indep	-0.101	Indep	-0.135^**^	Indep	-0.085
	(0.14)		(0.05)		(0.21)
Organ	0.027	Organ	-0.017	Organ	0.034^**^
	(0.10)		(0.35)		(0.04)
GDP	0.150^***^	GDP	0.146^***^	GDP	0.139^***^
	(0.00)		(0.00)		(0.00)
FDI	1.446^***^	FDI	1.450^***^	FDI	1.569^***^
	(0.00)		(0.00)		(0.00)
FIN	0.859	FIN	0.803	FIN	1.132^*^
	(0.14)		(0.17)		(0.05)
cons	-5.111^***^	cons	-5.119^***^	cons	-4.887^***^
	(0.00)		(0.00)		(0.00)
N	19141	N	19141	N	19141
Ind/Year	YES	Ind/Year	YES	Ind/Year	YES
AR2	0.674	AR2	0.675	AR2	0.677

Note: The table panel A, panel B and panel C respectively test the three moderating effects of matching degree of cash flow rights and control rights, supervision of major shareholders and willingness of subsidiaries to cooperate. The explained variable is total factor productivity (TFP), the explanatory variable is centralized allocation of decision rights (Cen), the moderating variables are matching degree of cash flow rights and control rights (PPD), supervision of major shareholders (Top1), willingness of subsidiaries to cooperate (SOP), and the control variables are company size (Size), asset-liability ratio (Lev), company age (Age), return of assets (Roa), cash flow (Cash), R&D intensity (Rd), independent director ratio (Indep), institutional shareholding ratio (Organ), regional economic level (GDP), foreign direct investment (FDI), and financial development level (FIN). Robust standard errors are presented in parentheses. ***, **, and * denote statistical significance at the 1%, 5%, and 10% levels, respectively.

#### 5.2.2. The supervision of major shareholders.

In China, large shareholders often have actual control of the group, and their interests are bound to the interests of the group. They not only have a strong incentive to promote enterprise transformation and upgrading to achieve steady growth in shareholder wealth, but also have the ability to promote enterprise transformation and upgrading by monitoring the self-interested behavior of subsidiaries. If so, it could be conjectured that the positive effect of the centralized allocation of decision rights on enterprise transformation and upgrading is more pronounced for groups with strong supervision of major shareholders. Following Wang et al. [45], we employ the shareholding ratio of the largest shareholder (Top1) to measure the supervision of major shareholders, and the larger the value, the stronger the supervision of major shareholders. Based on model (1), the interaction term of Cen and Top1 and the Top1 variable are added, and the baseline model is re-estimated. Panel B in Table 5 presents the estimation results. The coefficient on the interaction term is 0.003 and significantly positive, showing that the positive effect of the centralized allocation of decision rights on enterprise transformation and upgrading is more pronounced for groups with strong supervision of major shareholders, which is consistent with the above conjecture.

#### 5.2.3. The willingness of subsidiaries to cooperate.

Whether the governance advantages of the centralized allocation of decision rights can be effectively exerted depends to a certain extent on the willingness of subsidiaries to cooperate. Generally speaking, the stronger the willingness of subsidiaries to cooperate, the lower the inefficient investment level at the subsidiary level can be, and the more conducive it is to establishing and maintaining an internal capital market, thus enhancing the efficiency of resource allocation and knowledge transfer within the group, and ultimately facilitating enterprise transformation and upgrading. Following Zhu and Kong [25], we use subsidiary business scale (SOP), measured as the difference between consolidated statement operating income and parent company operating income divided by consolidated statement operating income, as a proxy variable for the willingness of subsidiaries to cooperate. Based on the model (1), the interaction term of Cen and SOP and the SOP variable are added, and the baseline model is re-estimated. Panel C in Table 5 presents the estimation results. The coefficient on the interaction term is 0.230 and significantly positive, suggesting that the positive effect of the centralized allocation of decision rights on enterprise transformation and upgrading is more pronounced for groups with a strong willingness of subsidiaries to cooperate, which is in line with the above analysis.

#### 5.2.4. Ownership type of the enterprise.

The difference of enterprise ownership type will directly affect the financing environment and governance mode of enterprises. The centralized allocation of decision rights helps enterprises to better respond to national policies and integrate various resources, form a unified strategic plan and action plan, and promote the transformation and upgrading of enterprises. Compared with state-owned enterprises, private enterprises are relatively limited in resource acquisition, and the promotion effect of centralized allocation of decision rights on enterprise transformation and upgrading will be reduced [46]. Foreign enterprises usually need to take into account the global strategy and the needs of the local market, with a more standardized and international management system, the decision-making process is often more complex. This may weaken the promotion effect of centralized allocation of decision rights of foreign enterprises on enterprise transformation and upgrading. In addition, there are differences in the governance concepts and experiences of enterprises with different ownership types. State-owned enterprises are usually controlled by the government, and their decision-making power is concentrated in higher-level management institutions or government departments to a certain extent to ensure the consistency and implementation of policies. State-owned enterprises may accumulate more experience about centralized decision-making to promote change in the long-term development, while private enterprises and foreign enterprises may need to pay more to adapt and adjust. This will lead to different effects of centralized allocation of decision rights on enterprise transformation and upgrading under different ownership types.

Therefore, this paper divides the samples into state-owned enterprise samples, private enterprise samples and foreign enterprise samples, and tests the impact of centralized allocation of decision rights on enterprise transformation and upgrading. Table 6 panel A, panel B and panel C are the test results of state-owned enterprise samples, private enterprise samples and foreign enterprise samples respectively. The results show that the Cen coefficients are all significantly positive. The regression coefficient of Cen in the state-owned enterprise sample and private enterprise sample are significantly positive at the level of 1%, while the regression coefficient of Cen in the foreign enterprise sample is only significantly positive at the level of 10%. Therefore, compared with foreign enterprise sample, the promotion effect of centralized allocation of decision rights on enterprise transformation and upgrading is more significant in the samples of state-owned enterprises and private enterprises. Furthermore, the regression coefficient of Cen in state-owned enterprise sample is greater than that in private enterprise sample. This shows that when other conditions remain unchanged, the conclusion of this paper are applicable to enterprises with different ownership types, but the influence degree is different.

**Table 6:** Grouping test of enterprise ownership types.

Variables	Panel A state-owned enterprises	Panel B private enterprises	Panel C foreign enterprises
TFP	TFP	TFP
Cen	0.152^***^	0.094^***^	0.118^*^
	(0.00)	(0.00)	(0.07)
Size	0.481^***^	0.465^***^	0.441^***^
	(0.00)	(0.00)	(0.00)
Lev	0.631^***^	0.784^***^	0.760^***^
	(0.00)	(0.00)	(0.00)
Age	0.114^***^	-0.001	0.021
	(0.00)	(0.88)	(0.36)
Roa	2.318^***^	1.886^***^	1.831^***^
	(0.00)	(0.00)	(0.00)
Cash	-0.084	-0.074	-0.013
	(0.41)	(0.13)	(0.92)
Rd	1.943^***^	-0.270	1.705^***^
	(0.00)	(0.29)	(0.00)
Indep	-0.060	-0.099	-0.165
	(0.61)	(0.28)	(0.43)
Organ	0.053	0.041^*^	0.096^*^
	(0.26)	(0.05)	(0.10)
GDP	0.124^***^	0.167^***^	0.040
	(0.00)	(0.00)	(0.37)
FDI	1.822^***^	0.948^**^	3.794^***^
	(0.00)	(0.03)	(0.00)
Fin	0.189	1.460^*^	-2.248
	(0.86)	(0.05)	(0.23)
cons	-5.356^***^	-5.222^***^	-3.406^***^
	(0.00)	(0.00)	(0.00)
N	6827	10906	1408
Ind/Year	YES	YES	YES
AR2	0.685	0.606	0.655

Note: The table panel A, panel B and panel C are the test results of three groups of samples: state-owned enterprise, private enterprise and foreign enterprise. The explained variable is total factor productivity (TFP), the explanatory variable is centralized allocation of decision rights (Cen), and the other control variables are company size (Size), asset-liability ratio (Lev), company age (Age), return of assets (Roa), cash flow (Cash), R&D intensity (Rd), independent director ratio (Indep), institutional shareholding ratio (Organ), regional economic level (GDP), foreign direct investment (FDI), and financial development level (FIN). Robust standard errors are presented in parentheses. ***, **, and *  denote statistical significance at the 1%, 5%, and 10% levels, respectively.

## 6. Discussion

Different from previous studies, this paper systematically analyzes the promotion effect of centralized allocation of decision rights on enterprise transformation and upgrading from the perspective of human capital level and capital allocation efficiency. The existing literature on the economic consequences of centralized allocation of decision rights mainly focuses on agency problem [19], enterprise efficiency [20], enterprise decision-making cost [21], resource mismatch [22] and enterprise innovation [23]. In particular, although this study is similar to Lou and Zhu’s research [23] on centralized allocation of decision rights and enterprise innovation, there are also essential differences. Specifically, first, although there is a correlation between the explained variable enterprise innovation studied by Lou and Zhu and the explained variable enterprise transformation and upgrading in this study, there are also differences. We usually think that enterprise innovation and enterprise transformation and upgrading are important events for enterprises. Although the goals of enterprise innovation and enterprise transformation and upgrading sometimes overlap, their core concerns and implementation paths are different. Enterprise innovation focuses more on the development of new products, new technologies and new processes, while enterprise transformation and upgrading focuses more on the adjustment of the overall structure and strategic re-planning. Enterprise innovation can be a means of enterprise transformation and upgrading, but the means of enterprise transformation and upgrading are not limited to enterprise innovation. On the contrary, there are many purposes of enterprise innovation, not necessarily for enterprise transformation and upgrading. Second, Lou and Zhu’s research mentioned the potential impact of centralized allocation of decision rights on enterprise innovation, but did not discuss its specific mechanism in depth. We analyze in detail how the centralized allocation of decision rights can promote the transformation and upgrading of enterprises by improving the level of human capital and the efficiency of capital allocation. Furthermore, in order to verify the validity of the theoretical analysis in this paper, we test the mediating effect of human capital level and capital allocation efficiency. The results show that the regression coefficient of Cen is significantly positive in the regression model of centralized allocation of decision rights (Cen) to human capital level (HCL). In the regression model of centralized allocation of decision rights (Cen) to enterprise inefficient investment (Absinv), the regression coefficient of Cen is significantly negative. This shows that the centralized allocation of decision rights does improve the human capital level and the capital allocation efficiency, thus promoting the enterprise transformation and upgrading.

We further enrich the research on the logical framework of the influence of centralized allocation of decision rights on enterprise transformation and upgrading under different special scenarios, such as the matching degree of cash flow rights and control rights, the supervision power of major shareholders and the willingness of subsidiaries to cooperate. From the parent-subsidiary level, we consider the preconditions for centralized allocation of decision rights of enterprise groups to give full play to their governance advantages, mainly focusing on the parent company’s tunneling motivation, the supervisory role of major shareholders, and the willingness of subsidiaries to cooperate with parent companies to complete major strategic tasks. The low hollowing-out motivation of the parent company means that the parent company is unlikely to seek personal gain by transferring resources or encroaching on the interests of subsidiaries, which is very important for the governance of group enterprises. The strong supervision function of major shareholders means that the internal governance structure of the enterprise is relatively sound, and the major shareholders can play an effective supervision function to ensure that the management follows the principles of transparency, legality and benefit to the overall interests of the group in the decision-making process. The strong willingness of subsidiaries to cooperate with the parent company means that there is a good communication and cooperation relationship between the parent company and subsidiaries, and subsidiaries are willing to support the strategic direction of the parent company, which is conducive to enhancing the overall interests of the group enterprises. The research results show that when the parent company’s tunneling motivation is low, the major shareholder’s supervision function is strong, and the subsidiary company’s willingness to cooperate with the parent company is strong, the centralized allocation of decision rights of the enterprise group is more likely to exert the governance advantage of focusing on major events, that is, the centralized allocation of decision rights has a stronger promotion effect on enterprise transformation and upgrading. In addition, considering that different types of enterprise ownership will have an impact on the governance effect of centralized allocation of decision rights, we further examine the heterogeneous effects of three types of ownership of state-owned enterprises, private enterprises and foreign enterprises on centralized allocation of decision rights and enterprise transformation and upgrading. The research results show that when other conditions remain unchanged, the conclusion of this paper are applicable to enterprises with different ownership types, but the influence degree is different. This not only emphasizes the applicability of the research conclusions, but also highlights the practical differences under different ownership types, which is of great significance for the promotion of theory and practical application.

The main deficiency of this paper lies in the measurement method of centralized allocation of decision rights in empirical tests. The allocation of decision rights is one of the core contents of enterprise internal governance mechanism, which involves the vertical distribution of decision rights between levels and the horizontal distribution between unit entities. The measurement method in this paper may not fully and accurately reflect the allocation of enterprise decision rights. In the future, we can continue to study the internal power allocation relationship of enterprise groups, explore more rigorous measurement indicators of decision rights allocation, and find more valuable research conclusions.

## 7. Conclusions

How to promote enterprise transformation and upgrading has increasingly attracted widespread academic attention. This paper advances academic understanding of whether and how the centralized allocation of decision rights within a group in emerging countries affects enterprise transformation and upgrading. Our findings evidence that the centralized allocation of decision rights has a positive effect on enterprise transformation and upgrading. Further analysis shows that enhanced human capital level and capital allocation efficiency are two mechanisms through which centralized allocation of decision rights affects enterprise transformation and upgrading. In addition, we find that the positive relation is more evident for groups with a high matching between cash flow rights and control rights, groups with strong supervision of major shareholders, and groups with a strong willingness of subsidiaries to cooperate.

Overall, this paper provides timely implications for enterprise groups and governments that intend to promote enterprise transformation and upgrading. To promote transformation and upgrading, enterprise groups should consider centralized management, and the government should appropriately guide enterprise groups to adopt a centralized management system and create convenient conditions for them. These findings have implications for other emerging economies, particularly those with large conglomerates and under the pressure of transformation and upgrading. Furthermore, when implementing centralized management, enterprise groups ought to account for particular circumstances. For example, when the matching degree of cash flow rights and control rights is high, the supervision of major shareholders is strong, and the willingness of subsidiaries to cooperate is strong, the centralized management may bring a better effect.

## Appendix 1

### Robustness tests

**Table:** 

Variables	Instrumental variable		PSM	Heckman	Cen with a lag of one year	Alternative measure
(1)	(2)	(3)	(4)	(5)	(6)
Cen	TFP	TFP	TFP	TFP	Patent
PGD	-0.565^***^					
	(0.00)					
Cen_PGD		0.595^***^				
		(0.00)				
Cen			0.053^***^	0.196^***^		0.645^***^
			(0.00)	(0.00)		(0.00)
IMR				7.000^***^		
				(0.00)		
L.Cen					0.151^***^	
					(0.00)	
Size	-0.015^***^	0.475^***^	0.469^***^	0.372^***^	0.466^***^	0.263^***^
	(0.00)	(0.00)	(0.00)	(0.00)	(0.00)	(0.00)
Lev	0.174^***^	0.657^***^	0.747^***^	1.520^***^	0.740^***^	-0.286^***^
	(0.00)	(0.00)	(0.00)	(0.00)	(0.00)	(0.00)
Age	-0.068^***^	0.078^***^	0.037^***^	-0.230^***^	0.049^***^	-0.079^***^
	(0.00)	(0.00)	(0.00)	(0.00)	(0.00)	(0.00)
Roa	0.395^***^	1.854^***^	2.087^***^	3.871^***^	2.049^***^	-0.430^***^
	(0.00)	(0.00)	(0.00)	(0.00)	(0.00)	(0.00)
Cash	-0.022	-0.056	-0.005	-0.145^***^	-0.061	-0.296^***^
	(0.23)	(0.21)	(0.94)	(0.00)	(0.18)	(0.00)
Rd	-0.133^*^	0.707^***^	0.189	-0.851^***^	0.553^***^	10.072^***^
	(0.09)	(0.00)	(0.45)	(0.00)	(0.00)	(0.00)
Indep	-0.178^***^	-0.026	-0.175^*^	-0.904^***^	-0.074	0.080
	(0.00)	(0.73)	(0.06)	(0.00)	(0.29)	(0.55)
Organ	0.070^***^	-0.002	0.036	0.380^***^	0.033^**^	-0.121^***^
	(0.00)	(0.93)	(0.11)	(0.00)	(0.05)	(0.00)
GDP	-0.044^***^	0.168^***^	0.150^***^	0.129^***^	0.152^***^	0.014
	(0.00)	(0.00)	(0.00)	(0.00)	(0.00)	(0.51)
FDI	0.439^***^	1.279^***^	1.168^***^	1.278^***^	1.469^***^	0.309
	(0.00)	(0.00)	(0.01)	(0.00)	(0.00)	(0.59)
FIN	0.404	0.417	0.531	1.188^**^	0.707	-0.740
	(0.10)	(0.49)	(0.50)	(0.03)	(0.23)	(0.49)
cons	0.903^***^	-5.657^***^	-5.242^***^	-8.047^***^	-5.167^***^	-5.113^***^
	(0.00)	(0.00)	(0.00)	(0.00)	(0.00)	(0.00)
N	18873	18873	10120	19141	18236	19141
Ind/Year	YES	YES	YES	YES	YES	YES
AR2	0.084	0.660	0.684	0.696	0.674	0.205
First-stage F-stat	/	362.384^***^	/	/	/	/

Note: Appendix 1 shows the robustness test results. The variables include total factor productivity (TFP), centralized allocation of decision rights (Cen), geographical distance of parent-subsidiary company (PGD), explanatory variables after instrumental variable estimation (Cen_PGD), inverse Mills Ratio (IMR), centralized allocation of decision rights with a lag of one year (L.Cen), company size (Size), asset-liability ratio (Lev), company age (Age), return of assets (Roa), cash flow (Cash), R&D intensity (Rd), independent director ratio (Indep), institutional shareholding ratio (Organ), regional economic level (GDP), foreign direct investment (FDI), and financial development level (FIN). In addition, because there are missing values when calculating the instrumental variable (PGD), the total samples in columns (1) and (2) are reduced. Robust standard errors are presented in parentheses. ***, **, and *  denote statistical significance at the 1%, 5%, and 10% levels, respectively.

## Appendix 2

### Balanced hypothesis testing

**Table:** 

Variables	Sample	Mean Difference		T-Value(p-Value)	Deviation(%)	
		Treatment Group	Control Group		%bias	%reduct
Size	Before matching	22.028	22.254	-12.52(0.000)	-18.1	
	After matching	22.028	22.052	-1.49(0.162)	-2	89.2
Lev	Before matching	0.406	0.416	-3.22(0.000)	-4.7	
	After matching	0.406	0.410	-1.40(0.160)	-0.2	56.8
Age	Before matching	2.040	2.254	-20.74(0.000)	-30	
	After matching	2.040	2.045	-0.45(0.654)	-0.7	97.8
Roa	Before matching	0.044	0.036	9.60(0.000)	13.9	
	After matching	0.044	0.044	0.08(0.939)	0.1	99.2
Cash	Before matching	0.005	0.006	-0.95(0.340)	-1.4	
	After matching	0.005	0.004	0.75(0.451)	1.1	18.9
Rd	Before matching	0.026	0.025	4.09(0.000)	5.9	
	After matching	0.026	0.026	1.45(0.148)	2.1	64.7
Indep	Before matching	0.372	0.377	-6.09(0.000)	-8.8	
	After matching	0.372	0.372	-0.04(0.967)	-0.1	99.4
Organ	Before matching	0.434	0.434	3.33(0.001)	4.8	
	After matching	0.434	0.440	-1.64(0.102)	-2.4	50.7
GDP	Before matching	11.085	11.122	-5.52(0.000)	-0.8	
	After matching	11.085	11.084	0.08(0.936)	0.1	98.6
FDI	Before matching	0.024	0.024	-2.35(0.019)	-3.4	
	After matching	0.024	0.024	-0.86(0.391)	-1.3	63
FIN	Before matching	0.009	0.008	2.48(0.013)	3.6	
	After matching	0.009	0.009	-1.03(0.302)	-1.5	57.8

Note: Appendix 2 details the results of the equilibrium hypothesis tests. The variables include company size (Size), asset-liability ratio (Lev), company age (Age), return of assets (Roa), cash flow (Cash), R&D intensity (Rd), independent director ratio (Indep), institutional shareholding ratio (Organ), regional economic level (GDP), foreign direct investment (FDI), and financial development level (FIN).

## Appendix 3

### Heckman selection model

**Table:** 

Panel A First Stage		Panel B Second Stage	
Variables	Cen	Variables	TFP
MCen	0.854^***^	Cen	0.196^***^
	(0.00)		(0.00)
Size	-0.022^***^	IMR	7.000^***^
	(0.00)		(0.00)
Lev	0.199^***^	Size	0.372^***^
	(0.00)		(0.00)
Age	-0.058^***^	Lev	1.520^***^
	(0.00)		(0.00)
Roa	0.466^***^	Age	-0.230^***^
	(0.00)		(0.00)
Cash	-0.014	Roa	3.871^***^
	(0.49)		(0.00)
Rd	-0.178^**^	Cash	-0.145^***^
	(0.03)		(0.00)
Indep	-0.199^***^	Rd	-0.851^***^
	(0.00)		(0.00)
Organ	0.083^***^	Indep	-0.904^***^
	(0.00)		(0.00)
Zbcc	0.040^***^	Organ	0.380^***^
	(0.00)		(0.00)
Asset_ratio	0.054^***^	GDP	0.129^***^
	(0.00)		(0.00)
Balance	-0.024^***^	FDI	1.278^***^
	(0.00)		(0.00)
Mshare	0.000^**^	FIN	1.188^**^
	(0.01)		(0.03)
cons	0.492^***^	cons	-8.047^***^
	(0.00)		(0.00)
N	19140	N	19141
Ind/Year	YES	Ind/Year	YES
AR2	0.077	AR2	0.696

Note: Appendix 3 Panel A and Panel B are the first and second stages of the Heckman selection model. The variables include total factor productivity (TFP), centralized allocation of decision rights (Cen), industry annual average with centralized allocation of decision rights (MCen), inverse Mills Ratio (IMR), company size (Size), asset-liability ratio (Lev), company age (Age), return of assets (Roa), cash flow (Cash), R&D intensity (Rd), independent director ratio (Indep), institutional shareholding ratio (Organ), regional economic level (GDP), foreign direct investment (FDI), and financial development level (FIN). The ratio of net fixed assets to total operating income (Zbcc), The ratio of the total assets of the parent company’s statements to the total assets of the consolidated statements (Asset_ratio), The ratio of the shareholding ratio of the 2nd to 5th largest shareholder to that of the largest shareholder (Balance), managerial ownership (Mshare). Robust standard errors are presented in parentheses. ***, **, and *  denote statistical significance at the 1%, 5%, and 10% levels, respectively.

## Appendix 4

### Heckman selection model supplementary results

**Table:** 

Variables	(1)	(2)	Variables	(3)	(4)	Variables	(5)	Variables	(6)
Cen	TFP	Cen	TFP	Cen	TFP
MCen	0.831^***^		PGD	-0.565^***^		Size	-0.022^***^	Cen	0.196^***^
	(0.00)			(0.00)			(0.00)		(0.00)
Cen		0.123^***^	Cen		0.111^***^	Lev	0.200^***^	IMR	9.022^***^
		(0.00)			(0.00)		(0.00)		(0.00)
IMR		1.779^***^	IMR		-0.730^***^	Age	-0.058^***^	Size	0.345^***^
		(0.00)			(0.00)		(0.00)		(0.00)
Size	-0.020^***^	0.443^***^	Size	-0.015^***^	0.475^***^	Roa	0.475^***^	Lev	1.750^***^
	(0.00)	(0.00)		(0.00)	(0.00)		(0.00)		(0.00)
Lev	0.170^***^	0.934^***^	Lev	0.174^***^	0.660^***^	Cash	-0.013	Age	-0.311^***^
	(0.00)	(0.00)		(0.00)	(0.00)		(0.52)		(0.00)
Age	-0.065^***^	-0.028	Age	-0.068^***^	0.077^***^	Rd	-0.17^9**^	Roa	4.395^***^
	(0.00)	(0.24)		(0.00)	(0.00)		(0.03)		(0.00)
Roa	0.401^***^	2.513^***^	Roa	0.395^***^	1.859^***^	Indep	-0.202^***^	Cash	-0.164^***^
	(0.00)	(0.00)		(0.00)	(0.00)		(0.00)		(0.00)
Cash	-0.017	-0.095^**^	Cash	-0.022	-0.057	Organ	0.084^***^	Rd	-1.273^***^
	(0.40)	(0.03)		(0.26)	(0.19)		(0.00)		(0.00)
Rd	-0.270^***^	0.287	Rd	-0.133	0.702^***^	Zbcc	0.040^***^	Indep	-1.139^***^
	(0.00)	(0.18)		(0.11)	(0.00)		(0.00)		(0.00)
Indep	-0.171^***^	-0.295^***^	Indep	-0.178^***^	-0.029	Asset_ratio	0.053^***^	Organ	0.482^***^
	(0.00)	(0.00)		(0.00)	(0.68)		(0.00)		(0.00)
Organ	0.077^***^	0.124^***^	Organ	0.070^***^	-0.000	Balance	-0.024^***^	GDP	0.121^***^
	(0.00)	(0.00)		(0.00)	(1.00)		(0.00)		(0.00)
GDP	-0.046^***^	0.097^***^	GDP	-0.044^***^	0.167^***^	Mshare	0.000^***^	FDI	1.376^***^
	(0.00)	(0.00)		(0.00)	(0.00)		(0.01)		(0.00)
FDI	0.502^***^	2.036^***^	FDI	0.439^***^	1.289^***^			FIN	1.278^**^
	(0.00)	(0.00)		(0.00)	(0.00)				(0.02)
FIN	0.706^***^	1.583^**^	FIN	0.404	0.428				
	(0.01)	(0.01)		(0.11)	(0.47)				
cons	1.024^***^	-5.392^***^	cons	0.963^***^	-4.972^***^	cons	0.497^***^	cons	-8.857^***^
	(0.00)	(0.00)		(0.00)	(0.00)		(0.00)		(0.00)
N	19141	19141	N	18873	18873	N	19141	N	19141
Ind/Year	YES	YES	Ind/Year	YES	YES	Ind/Year	YES	Ind/Year	YES
AR2	0.069	0.674	AR2	0.084	0.674	AR2	0.074	AR2	0.698

Note: Appendix 4 is the Heckman selection model supplementary results. First, we use the industry annual average with centralized allocation of decision rights (MCen) as the instrumental variable in the first stage of the Heckman selection model, and the control variables are consistent with the benchmark model (1). The regression results are shown in columns (1) and (2). Second, we use the geographical distance of the parent-subsidiary company (PGD) as an instrumental variable in the first stage of the Heckman selection model, and the control variables are consistent with the benchmark model (1). The regression results are shown in columns (3) and (4). Third, we delete the instrumental variable in the first stage regression of the Heckman selection model, and then estimate the model. The regression results are shown in columns (5) and (6). The variables include total factor productivity (TFP), centralized allocation of decision rights (Cen), industry annual average with centralized allocation of decision rights (MCen), geographical distance of parent-subsidiary company (PGD), inverse Mills Ratio (IMR), company size (Size), asset-liability ratio (Lev), company age (Age), return of assets (Roa), cash flow (Cash), R&D intensity (Rd), independent director ratio (Indep), institutional shareholding ratio (Organ), regional economic level (GDP), foreign direct investment (FDI), and financial development level (FIN). The ratio of net fixed assets to total operating income (Zbcc), The ratio of the total assets of the parent company’s statements to the total assets of the consolidated statements (Asset_ratio), The ratio of the shareholding ratio of the 2nd to 5th largest shareholder to that of the largest shareholder (Balance), managerial ownership (Mshare). Robust standard errors are presented in parentheses. ***, **, and * denote statistical significance at the 1%, 5%, and 10% levels, respectively.
